# Editorial: Introduction to the Presentations at the Factor XI and the Contact System SSC Session of the ISTH, Montpellier, France, May 27, 2016

**DOI:** 10.3389/fmed.2017.00140

**Published:** 2017-08-24

**Authors:** Alvin H. Schmaier, Joost C. M. Meijers

**Affiliations:** ^1^Department of Internal Medicine, Division of Hematology and Oncology, Case Western Reserve University, Cleveland, OH, United States; ^2^Department of Plasma Proteins, Sanquin Research, Amsterdam, Netherlands; ^3^Department of Experimental Vascular Medicine, Academic Medical Center, University of Amsterdam, Amsterdam, Netherlands

**Keywords:** coagulation, contact system, factor XII, factor XI, prekallikrein, high molecular weight kininogen, bradykinin, kallikrein–kinin system

**Editorial on the Research Topic**

**Introduction to the Presentations at the Factor XI and the Contact System SSC Session of the ISTH, Montpellier, France, May 27, 2016**

The present volume comprises some of the oral presentations that took place in the FXI and the Contact Activation Subcommittee on Standards of Coagulation in the Montpellier, France meeting on May 27, 2016. The assembly of articles is from participants who chose to submit their work. The cohesive bond that brings these manuscripts together is the active participation in the subcommittee’s program. There are six manuscripts that were assembled for this e-book.

The first two presentations address the business of the subcommittee, standardization. The first manuscript presented was by Rosén of Molndal, Sweden. Dr. Rosen applied a commercial assay for Factor XIa (FXIa) that employs an activated Factor X readout of hydrolysis of a chromogenic substrate. The assay provides Factors IX, VIII, and X. FXI as well as Factor XII (FXII) and prekallikrein activation in this assay are initiated by contact activation with a commercial aPTT reagent. Further, the stability of the assay may be improved by the use of MES-BSA buffer.

The second presentation was by Wilmot et al. of the National Institute for Biological Standards and Controls, Potters Bar, Hertfordshire, UK, on the establishment of an international standard for human plasma Factor XI. Twenty-nine laboratories participated in the functional assay; 11 laboratories participated in the antigen assay. The plasma standard was established to have a functional activity value (FXI:C) of 0.71 U/ml and an antigen value (FXI:Ag) of 0.78 IU/ml.

The next series of presentations focused on mechanisms associated with contact proteins. Jukema et al. of Utrecht, Netherlands, presented some work in progress on FXII processing in a non-canonical manner during inflammatory reactions. The authors raise the hypothesis that non-contact system enzymes, e.g., plasmin or elastase, prime zymogen FXII to produce a βFXII that then gets activated to βFXIIa or the so-called FXII fragment. These processes may be active during inflammatory processes such as Gram-negative sepsis, anaphylaxis, and neuroinflammatory disorders like multiple sclerosis.

Schmaier presented a review of his recent work characterizing mechanisms for reduced thrombosis observed in genetics deletions of proteins of the contact system. Although most agree that *f12^−/−^* mice have reduced contact activation for their mechanism for reduced thrombosis risk, investigations in the Schmaier laboratory indicate that *klkb1^−/−^* (prekallikrein deficient) and *bdkrb2^−/−^* (bradykinin B2 receptor deficient) mice have a non-contact activation-mediated mechanism. Studies show that both these animals have reduced vessel wall tissue factor arising from elevated prostacyclin levels to reduce their thrombosis risk. Prostacyclin levels in *klkb1^−/−^* mice are less than *bdkrb2^−/−^* who also have a selective platelet GPVI and spreading defect.

Weitz and Fredenburgh of Hamilton, ON, Canada presented their thoughts of targeting Factors XI or XII as safer choices to prevent thrombosis. In this perspective, rationales for inhibiting FXIa over FXIIa and *vice versa* are presented; advantages of one versus the other are discussed; and strategies for inhibition of either also are presented. Further, initial human trials of FXI knockdown are delineated. Last the authors discuss the potential unmet needs in anticoagulation and antithrombotic therapy by targeting FXII or FXI. These novel agents provide new opportunities for improved patient care.

Finally, Simão and Feener provide a detailed review of the contact system and their hypothesis how plasma kallikrein and its product of high-molecular-weight kininogen cleavage, bradykinin, may contribute to hemorrhage. The Feener lab has presented data to suggest that plasma kallikrein cleaves collagen to reduce platelet aggregation, and liberated bradykinin has potential to elevate prostacyclin and tissue plasminogen activator that could contribute to bleeding.

In sum, these articles present in part some of the diverse and active research in the FXI, FXII, and contact activation system. This field has developed new research avenues that should lead to novel therapies for presently unrealized targets in this increasingly important area.

## Author Contributions

Each author contributed an equal share to this manuscript.

## Conflict of Interest Statement

The authors declare that the research was conducted in the absence of any commercial or financial relationships that could be construed as a potential conflict of interest.

